# Identification of the characteristics of infiltrating immune cells in pulpitis and its potential molecular regulation mechanism by bioinformatics method

**DOI:** 10.1186/s12903-023-03020-z

**Published:** 2023-05-13

**Authors:** Jing Wang, Junxia Qiao, Lili Ma, Xin Li, Chengshi Wei, Xiufen Tian, Kun Liu

**Affiliations:** 1 Department of Stomatology, Liaocheng People’s Hospital, 67 Dongchang West Road, Liaocheng, Shandong 252000 P.R. China; 2 Department of Endodontics, Liaocheng People’s Hospital, Liaocheng, Shandong 252000 P.R. China

**Keywords:** Pulpitis, Immune cell infiltration, Immune-related differentially expressed genes, Signal pathways, Bioinformatics analysis

## Abstract

**Objective:**

The inflammation of dental pulp will also trigger an immune response. The purpose of this study is to demonstrate the immune cell’s function and explore their regulatory molecules and signal pathways in pulpitis.

**Method:**

The CIBERSORTx method was used to quantitatively analyze 22 types of immune cells infiltrating in the GSE77459 dataset of dental pulp tissues. The immune-related differential genes (IR-DEGs) were further screened and enriched for the GO and KEGG pathways. Protein–protein interaction (PPI) networks were constructed and the hub IR-DEGs were screened. Finally, we constructed the regulatory network of hub genes.

**Results:**

The GSE77459 dataset screened 166 IR-DEGs and was enriched for three signal pathways involved in pulpitis development: chemokine signaling, TNF signaling, and NF-κB signaling. Significant differences in immune cell infiltration were observed between normal and inflamed dental pulp. The proportions of M0 macrophages, neutrophils, and follicular helper T cells were significantly higher than that of the normal dental pulp, while the proportions of resting mast cells, resting dendritic cells, CD8 T cells, and monocytes were significantly lower. The random forest algorithm concluded that M0 macrophages and neutrophils were the two most important immune cells. We identified five immune-related hub genes IL-6, TNF-α, IL-1β, CXCL8, and CCL2. In addition, IL-6, IL-1β, and CXCL8 are highly correlated with M0 macrophages and neutrophils, and the five hub genes have many shared regulatory molecules: four miRNAs and two lncRNAs, three transcription factors.

**Conclusion:**

Immune cell infiltration plays an important role in pulpitis among which M0 macrophages and neutrophils are the most significant immune cells. IL-6, TNF-α, IL-1, CXCL8, and CCL2 may be essential molecule of the immune response regulation network in pulpitis. This will help us understand the immune regulatory network in pulpitis.

## Introduction

Dental tissue may suffer severe damage due to various effects such as dental caries or trauma [1]. Once the dentin layer is endangered, the dental pulp will start its complex defense function, and at the same time, the microbial-related humoral and cellular immune mechanisms are activated, leading to the initial reversible local inflammation [2]. Human teeth have the potential for immune defense before eruption; In the initial stage of dental caries infection, the immune response mediated by class-II-expressing cells is activated in human dental pulp [3]. The dental pulp is an active homeostatic tissue that resists external stimuli during tooth decay by activating immunocompetent cells involved in innate and adaptive responses [4]. The dental pulp is a complex tissue composed of many types of cells, and its dentin-pulp complex acts as a barrier, in which odontoblasts and immune cells are strategically distributed to prevent the invasion of pathogens. There were significant differences in the distribution of odontoblasts, macrophages, and neutrophils and morphological changes between the irreversible pulpitis and the healthy control [5, 6]. Dental pulp tissue produces proinflammatory cytokines and chemokines upon recognition of pathogens, including chemokine (C–C motif) ligand 2 (CCL2), CCL5, CCL7, chemokine (C-X-C motif) ligand 8 (CXCL 8), and CXCL10, which regulate immune cell recruitment and activation to coordinate immune responses [4, 7]. In inflamed human dental pulp, miR-150 and miR506 are significantly upregulated. Their overexpression activates the immune system and protects against inflammatory responses [8]. A key function of SIRT1 in HDPCS relies on its ability to mediate LPS- and heat-induced expression of immunity and defense genes [9]. Understanding the immune cell and molecular signals associated with the immune response during pulpitis is important.

Recent studies have shown a correlation between pulpitis susceptibility and gene expression using bioinformatics. For example, a novel lncRNA, PVT1 that mediates the regulation mechanism of ceRNA in the pathogenesis of pulpitis has been screened using bioinformatics analysis and also proven by experiments [10]. In addition, other researchers also analyzed the hub regulatory genes or differential lncRNA and its transcription factors involved in pulpitis using comprehensive bioinformatics analysis [11–13]. Thus, bioinformatics analysis of gene expression profiles may be useful for screening new biomarkers of pulpitis or for understanding the molecular mechanism of pulpitis. Bioinformatics technology can also predict the infiltrated immune cells in tissues through gene expression in microarray or sequencing datasets. Through a literature search, we didn't find any bioinformatics analysis about the infiltration of immune cells in the inflamed pulp tissue and the genes, cytokines, and signal pathways that might be involved.

Consequently, the purpose of this study was to employ bioinformatics analysis of gene expression synthesis (GEO) microarray dataset to investigate the characteristics of immune cell engaged in the immune reaction in pulpitis as well as potential immune-related hub genes and signaling pathways. Explaining the difference in immune infiltrated between inflamed and normal dental pulp tissue and the possible mechanisms of molecular regulatory networks will aid to understand the development of pulpitis.

## Methods

### Data collection

The GEO database provided the matrix files for the GSE77459 dataset using the GPL17692 platform, which contained 6 normal pulp tissues and 6 inflamed pulp tissues and the information of GSE77459 dataset is shown in Table 1. Additionally, 2483 immunity-related genes (IRGs) were retrieved from ImmPort. An Ethics Committee at Liaocheng People's Hospital in Shandong Province, China, approved this study.

**Table 1:** The information of GSE77459 dataset

GEO	Study type	platform	Tissue	n (normal)	n (inflamed)	PubMed ID
GSE77459	Expression profiling by array	GPL17692	Dental pulp tissue	6	6	33011745

### Assessment of immune cell subtype distribution

CIBERSORTx is an algorithm for deconvoluting immune cells that can analyze any subtype of immune cell and accurately quantify the characteristics of the resulting immune cells. This study calculated the immune cell composition between normal and inflamed pulp tissue using CIBERSORTx. We used the LM22 signature and 1000 permutations to calculate the fractions of 22 immune cell types. Then fully analyze and visualize the correlation heatmap, proportion, and different expressions between immune cells and samples.

### Identification of IR-DEGs in pulpitis that are differentially expressed

The "limma" package of R was used to analyze the gene expression matrix of the GSE77459 dataset, which contains DEGs between normal pulp tissues and inflamed pulp tissues (limma, https://bioconductor.org/packages/limma/). Then, For the selection of DEGs, |log2FC| >2 and a false discovery rate (FDR) of 0.05 were used as significance indicators. For the subsequent analysis, we combined the DEGs with 2484 IRGs to obtain overlapping genes.

### An analysis of IR-DEGs' functional enrichment and pathway analysis

Gene Ontology (GO) annotation was used to explain the biological significance of IR-DEGs. Kyoto Encyclopedia of Genes and Genome (KEGG) was used to investigate the signaling pathways of these IR-DEGs (www.kegg.jp/kegg/kegg1.html). The clusterProfiler function of the R package was used to perform analyses of GO terms and KEGG pathways [14]. *P*<0.05 and count≥10 were used as cutoff values. The enrichment results are displayed using bar and chord graphs in the ClusterProfiler package.

### Analysis of gene expression using the PPI network for the IR-DEGs

A functional protein association network based upon core factors was assessed using the STRING database. A PPI network of IR-DEGs was constructed using STRING. We used Cytoscape visualization with a confidence score greater than 0.9 and unconnected genes were hidden. An application of Molecular Complex Detection (MCODE) on Cytoscape was used to examine functional clusters of genes in the PPI network using degree cutoffs of 2, node scores of 0.2, k cores of 3, and a maximum depth of 100 as filters. The module with established scores >5 was screened out using CytoHubba in Cytoscape, which found the Top10 nodes in six ways, and screened the hub genes using the intersection.

### Prediction and construction of hub gene regulatory network

Based on human transcription factor information, hub genes' transcription factors were predicted (miRNet, https://www.mirnet.ca/miRNet/upload/GeneUploadView.xhtml), and then Cytoscape software was used to visualize transcription factors (TFs) and hub genes in the regulatory network.

#### Statistical analysis

Most statistical analyses are performed using R (version 4.0), which includes the affy and impute packages for data standardization, the limma package for difference analysis, and the ggplot2 package for data visualization. *Wilcoxon tests* were used to compare the data from various groups. Spearman's rank correlation was used to determine the correlation between immune cells and IR-DEGs. Significant differences were considered at *p < 0.05, **p < 0.01, ***p < 0.001, and ****p < 0.0001.

## Results

### Characteristics and differences of immune cells in the normal and inflammatory dental pulp tissue

**Fig. 1:**
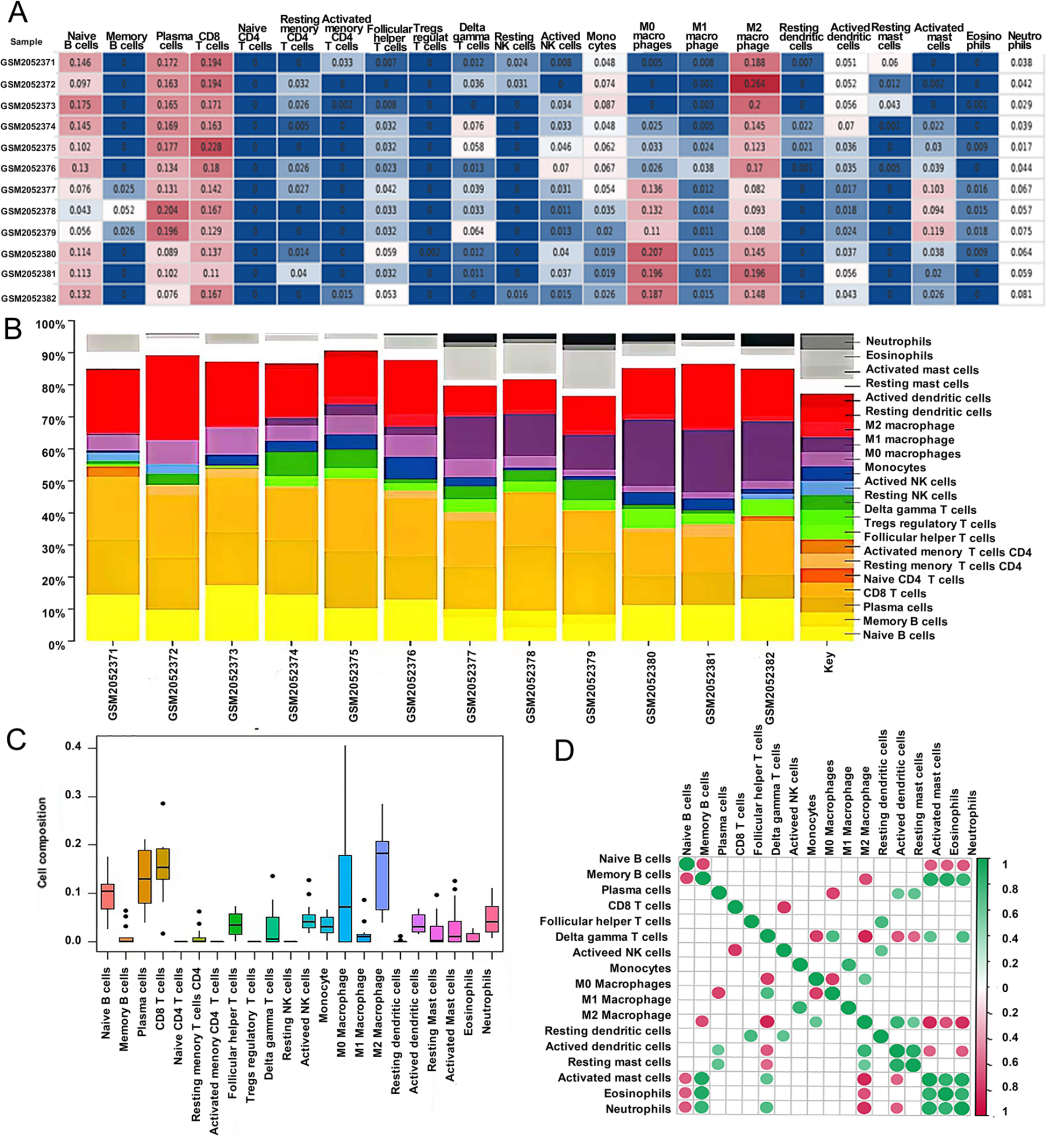
Evaluation and visualization of immune cell infiltration. **A** Results of the CIBERSORTx analysis. The heavier the color, the more significant the difference. **B** Percentage of 22 immune infiltrating cells in each sample. **C** The composition of immune cells in dental pulp tissue. **D** Correlation of immune infiltrating cells. Values representing the correlation coefficient between the immune cells (range 1 to 1) are shown in the upper right. Immune cells with higher, lower, and the same associated levels were shown as green, purple, and white, respectively. Significant *P*-values for the correlation between the immune cells are shown in the lower left

**Fig. 2:**
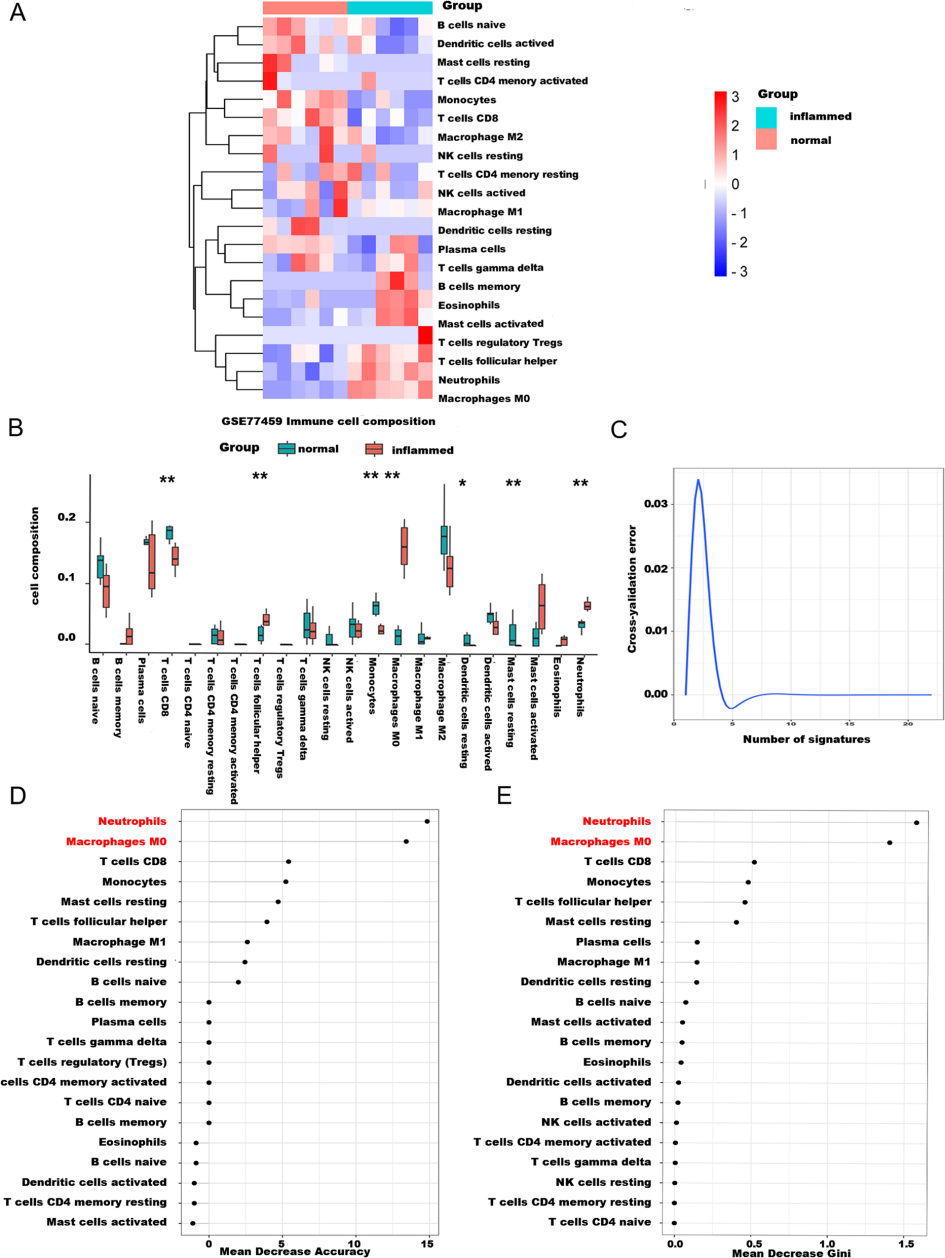
**A** Heatmap of immune cell score, with different colors representing expression trends in different samples. **B** Comparison of the difference between immune infiltrating cells in pulpitis and healthy samples. **C**-**E** Random Forest was used to examine the differences in immune cell infiltration in normal and pulpitis. The key immune cell types related to pulpitis were determined by (**D**) average descending accuracy and (**E**) average descending Gini coefficient

We first identified the profile of 22 infiltrating immune cells in pulpitis, and pulpitis samples were compared to normal samples using the CIBERSORTx algorithm. The results showed that naïve B cells, plasm cells, CD8 T cells, M0 macrophages, M2 macrophages, and neutrophils were the main immune cells that infiltrated the dental pulp tissue (Fig. 1A). As seen in Fig. 1B and C, the 22 infiltrating immune cells were distributed differently between the two groups. The correction between immune cells in pulpitis was further revealed, and the results are shown in Fig. 1D. Inflamed pulp tissue showed a significant difference in immune cell infiltration from normal pulp tissue (Fig. 2A). Following that, we used the Wilcoxon test to identify immune cells that differ between normal and inflamed pulp tissues (Fig. 2B). A total of 7 immune cells were significantly different in GSE77459 dataset. For example, the proportion of M0 macrophages, neutrophils, and follicular helper T cells was significantly higher than that in the normal pulp tissue, and the proportion of resting mast cells, resting dendritic cells, CD8 T cells, and monocytes were significantly fewer than that in normal pulp tissue. To identify disease-critical immune cell types, a random forest algorithm was applied, with M0 macrophages and neutrophils ranked at the top by two feature weights (Fig. 2C-E).

### Determination of differentially expressed immune-related genes (IR-DEGs)

**Fig. 3:**
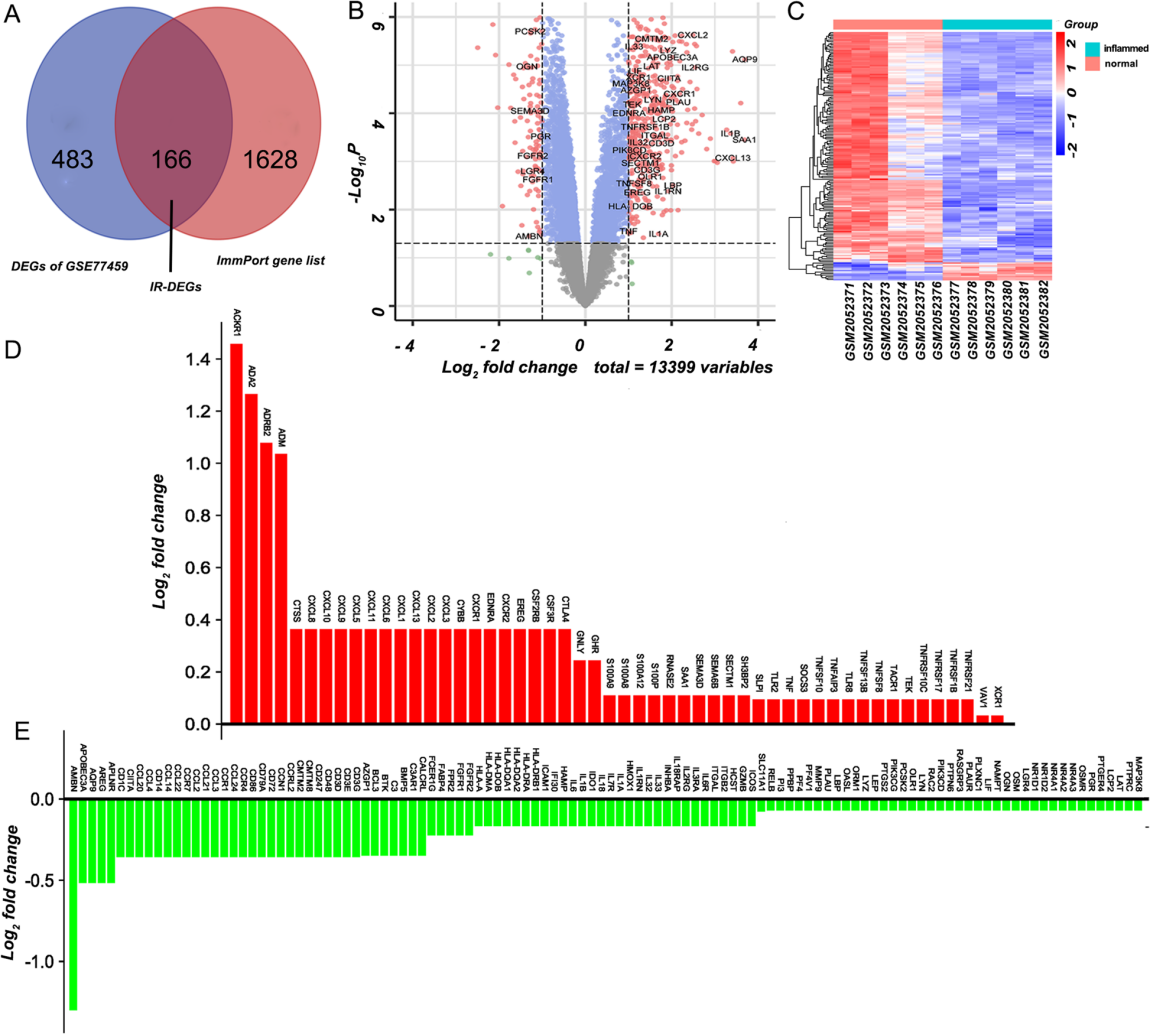
**A** Venn diagram was conducted to obtain the IR-DEGs screened by the ImmPort database. **B** Volcano map of all DEGs in the inflamed group and the control group Mark the IR-DEGs. **C** Heatmap of IR-EDGs in the inflamed group and the control group. **D** and **E** Log2FC value ranks differentially expressed genes as up-regulated (red) and down-regulated (green)

We performed the DEGs analysis between normal and inflamed pulp tissue samples and identified 649 DEGs, of which 138 genes were significantly upregulated and 511 genes were significantly downregulated in inflamed pulp tissues compared to normal pulp tissues. To further identify the DEGs associated with immunity, we intersected the 649 DEGs of the GSE77459 dataset with 2483 genes from the ImmPort database (Fig. 3A). The 166 IR-DEGs obtained are presented in Fig. 3B by a volcanic map, and with the results of cluster thermogram (Fig. 3C) showed that these differentially expressed genes could distinguish the samples of normal pulp group and inflammatory pulp group. The intriguing result is that numerous members of the CXC family, CCL family, IL family, and TNF family are differentially expressed, as indicated by the value of Log2FC value in our analysis of the DEGs (Fig. 3D and E).

### Analysis of IR-DEGs for functional enrichment

**Fig. 4:**
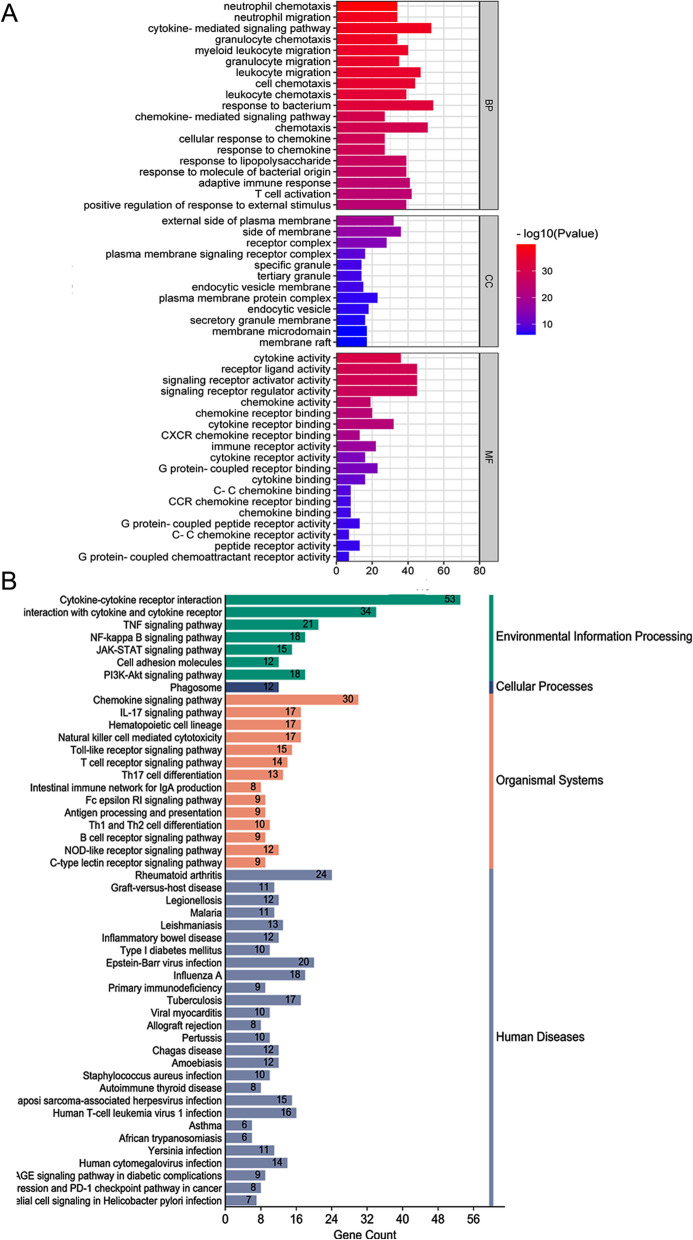
**A** GO enrichment of IR-DEGs. The bar graph shows the top processes enriched by IR-DEGs in BPs, CCs and MFs. **B** KEGG enrichment analysis of IR-DEGs. These pathways are further divided into four categories: environmental information processing, cell process, organism system, and human disease

**Fig. 5:**
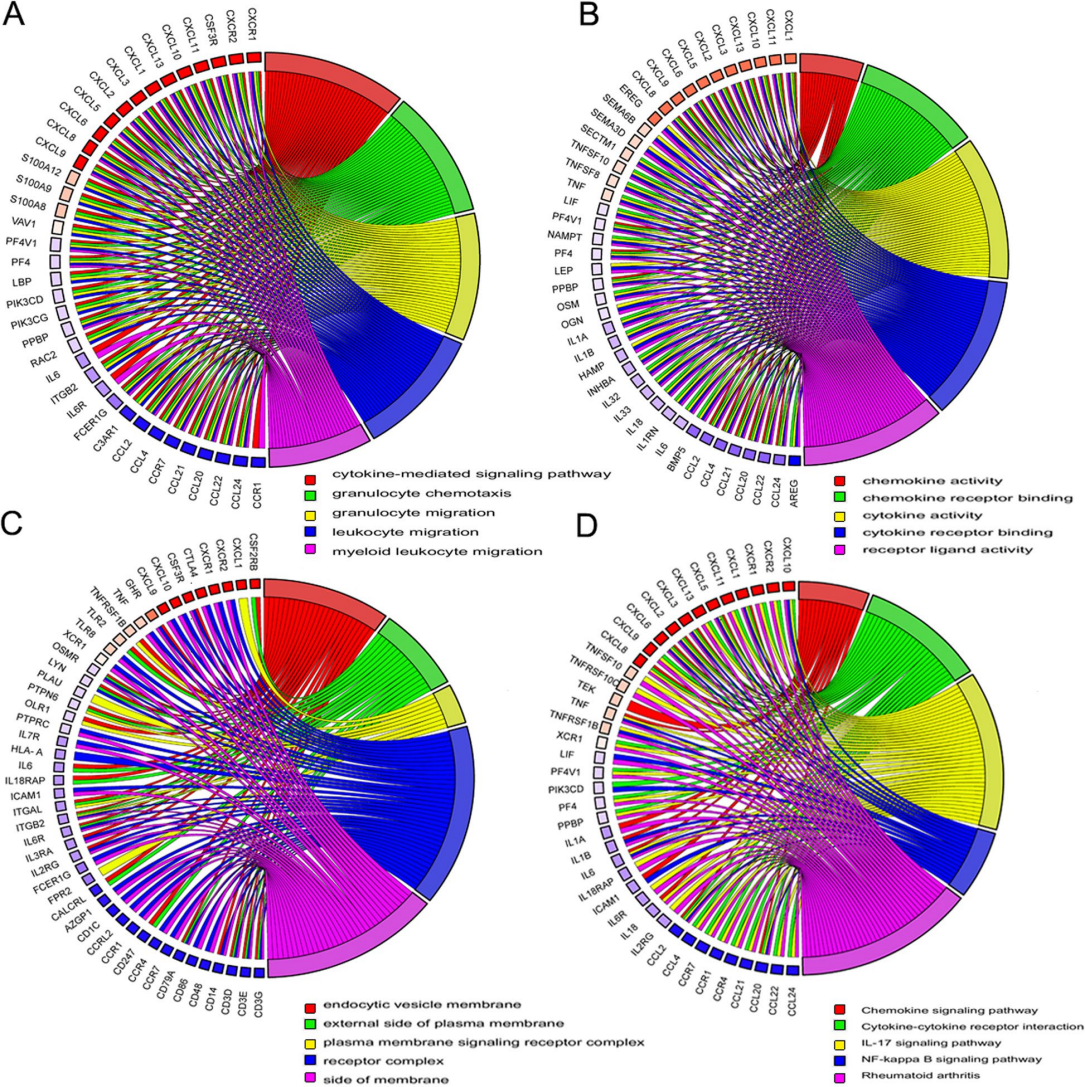
Chords from GO and KEGG analyses, the first 5 terms are shown as chords. **A** The chord plot of biological process (BPs) **B** The chord plot of molecular function (MFs) (**C**). The chord plot of Cellular component (CCs) (**D**). The chord plot of Kyoto encyclopedia of Genes and Genomes (KEGGs)

A GO and KEGG pathway analysis of IR-DEGs was performed to demonstrate their biological functions and pathways. As expected, IR-DEGs were mainly enriched in neutrophil chemotaxis, granulocyte chemotaxis, cytokine-mediated signaling pathway, neutrophil migration, myeloid leukocyte migration, and granulocyte migration in the BPs, cytokine activity, signaling receptor activator activity in the CCs, receptor-ligand activity, external side of the plasma membrane in the MFs (Fig. 4A). ClusterProfiler's centplot function displays IR-DEGs that are enriched in the Top 5 processes with the lowest *P* values for BPs, CCs, and MFs (Fig. 5A-C). ClusterProfiler's enrichKEGG function enriches KEGG pathway genes, referring to the human genome, and these genes are enriched in Cytokine-cytokine receptor interaction, Viral protein interaction with cytokine and cytokine receptor, Rheumatoid arthritis, Chemokine signaling pathway, TNF signaling pathway, and nuclear factor-κB (NF-κB) signaling pathway (Fig. 4B). The chord graph is used to enrich IR-DEGs in the Top5 processes of KEGG's minimum p-value (Fig. 5D).

### Identification of immune-related hub genes in the inflamed pulp tissue

**Fig. 6:**
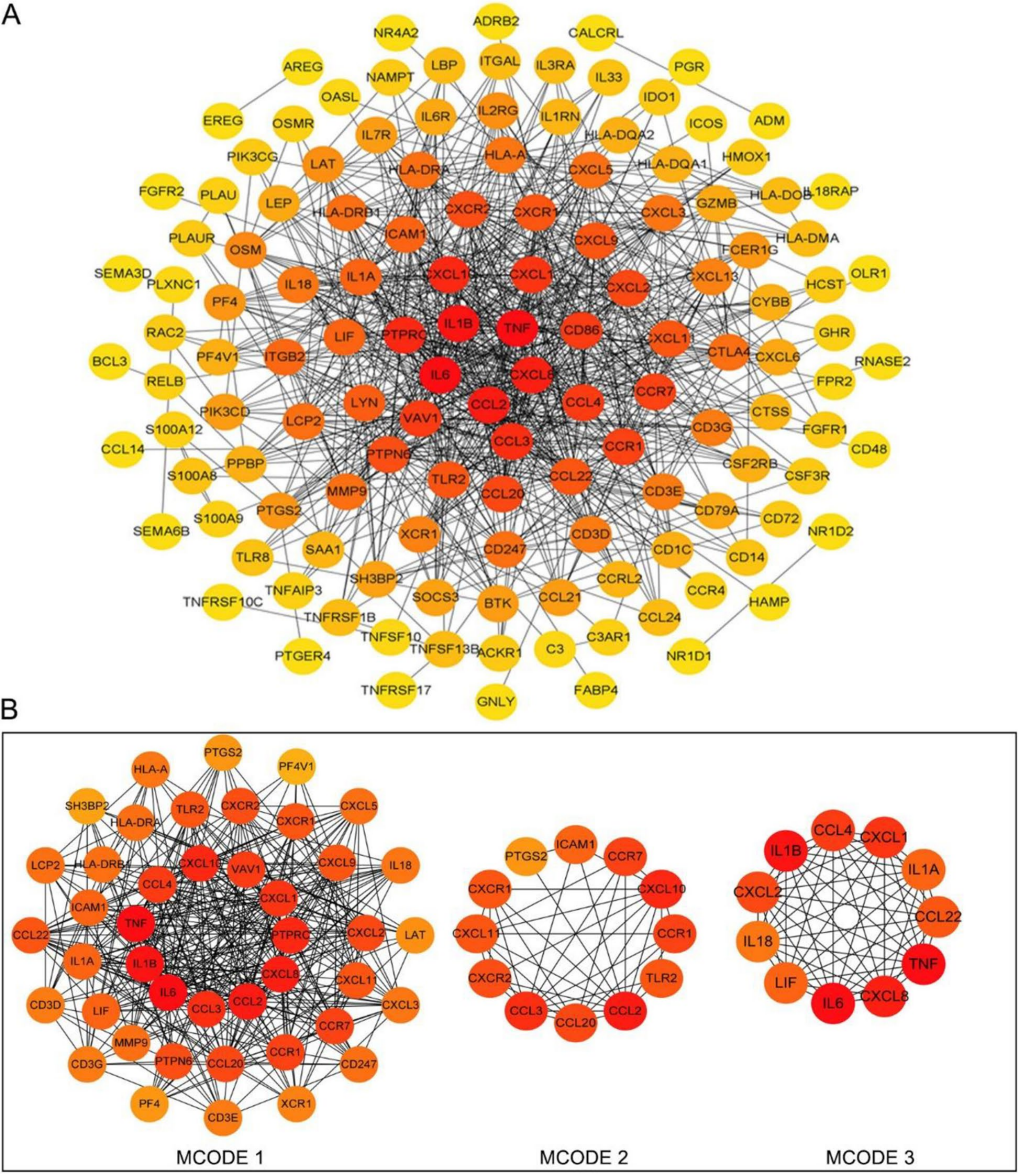
The PPI network of IR-DEGs and the identifying of hub genes. **A** The STRING database is used to construct the PPI network of IR-DEGs. The stronger the relationship between nodes, the heavier the color. **B** The top 3 node gene clusters with the highest scores were constructed by the MCODE plug-in

One hundred sixty-six IR-DEG proteins were analyzed using the STRING database to construct a PPI network of their interactions (Fig. 6A). We use the MCODE plug-in in Cytoscape to build functional modules, creating three modules with > 2 scores (Fig. 6B). Using the CytoHubba plug-in in Cytoscape, the first 10 hub genes were screened. There were 5 genes out of all 5 methods, i.e., IL-6, TNF, IL-1β, CXCL8, and CCL2 (Table 2). Based on the retrieved GPL117692 platform information and previous literature, TNF was confirmed to be TNF-α, which is our general gene symbol and hereinafter referred to as TNF-α.

**Table 2:** Top 10 IR-DEGs by 7 topological analysis methods of CytoHubba

Stress	Radiality	MCC	MNC	EPC	Closeness	Degree
IL6	IL6	CCL2	TNF	TNF	IL6	TNF
TNF	TNF	CXCL8	IL6	IL6	TNF	IL6
PTPRC	PTPRC	CXCL10	IL1B	CCL2	IL1B	IL1B
CD86	IL1B	CCL3	CCL2	CXCL10	PTPRC	CCL2
IL1B	CXCL8	CXCL1	CXCL8	IL1B	CXCL8	CXCL8
TLR2	CD86	CXCL2	PTPRC	CXCL8	CCL2	PTPRC
ITGB2	CCL2	CCL4	CXCL10	CCL3	CXCL10	CXCL10
VAV1	TLR2	IL1B	CCL3	CXCL1	CD86	CCL3
CXCL8	CXCL10	TNF	CXCL1	CCL20	CCL3	CXCL1
CCL2	CTLA4	IL6	CD86	CCR7	TLR2	CD86

### The correlation between immune infiltration cells and hub genes expression

**Fig. 7:**
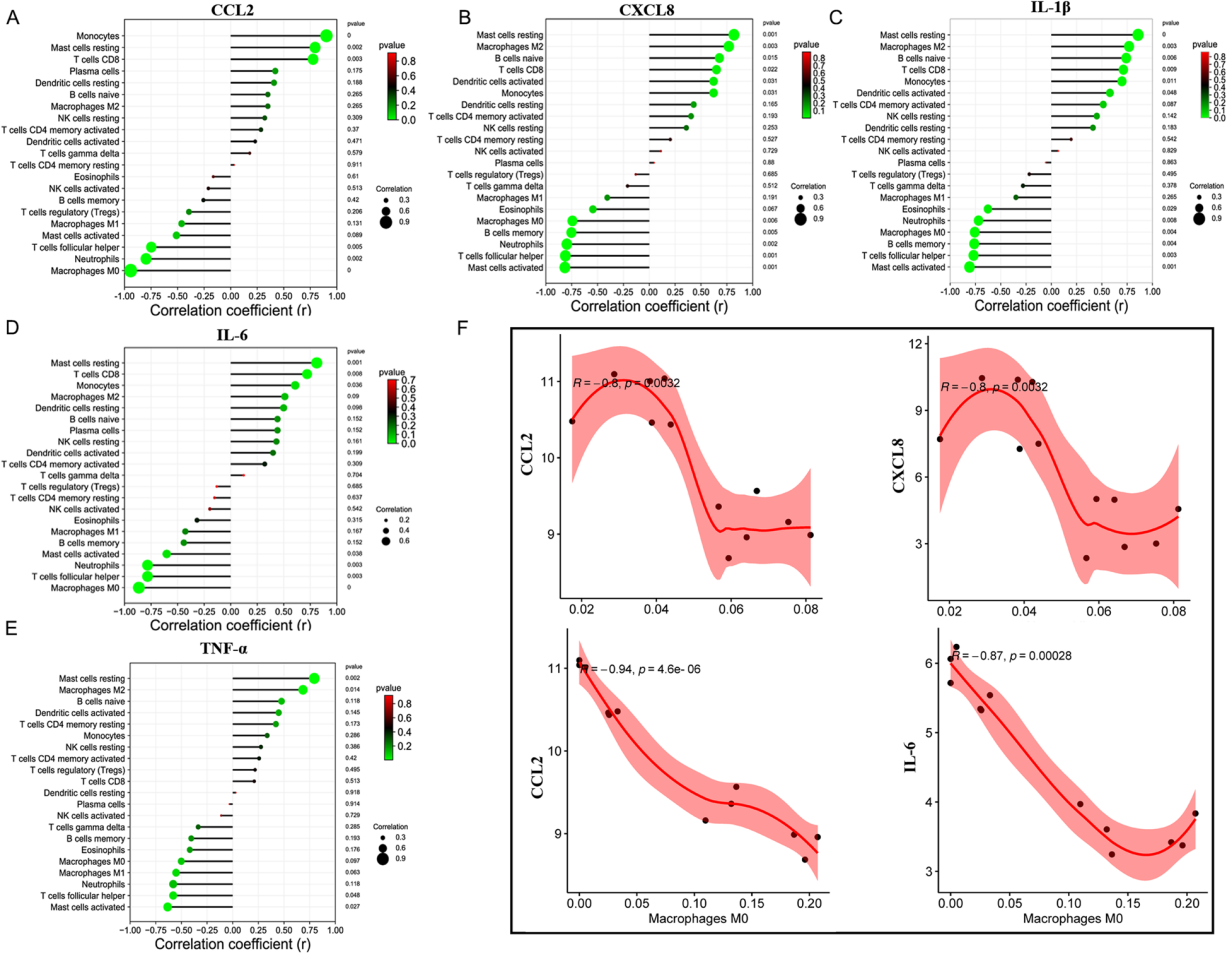
Correlation between hub genes and infiltrating immune cells. **A** Correlation between CCL2 and infiltrating immune cells. **B** Correlation between CXCL8 and infiltrating immune cells. **C** Correlation between IL1B and infiltrating immune cells. **D** Correlation between IL6 and infiltrating immune cells. **E** Correlation between TNF and infiltrating immune cells. **F** Spearman’s correlation between hub IR-DEGs and M0 macrophage and neutrophils

As shown in Fig. 7A-E, the infiltration levels of monocytes, resting mast cells, and CD8 T cells were positively correlated with CCL2 expression; the infiltration levels of follicular helper T cells, neutrophils, and M0 macrophages were negatively correlated with CCL2 expression. The infiltration levels of resting mast cells, M2 macrophages, naïve B cells, CD8 T cells, and activated dendritic cells, and monocytes were positively correlated with CXCL8 expression; the infiltration levels of memory B cells, neutrophils, follicular helper T cells, and activated mast cells were negatively correlated with CXCL8 expression. The infiltration levels of resting mast cells, M2 macrophages, naïve B cells, CD8 T cells, monocytes, and activated dendritic cells were positively correlated with IL-1β expression; The infiltration levels of M0 macrophages, memory B cells, follicular helper T cells, and activated mast cells were negatively correlated with IL-1β expression. The infiltration levels of resting mast cells, CD8 T cells, and monocytes were positively correlated with IL-6 expression; the infiltration levels of activated mast cells, neutrophils, and follicular helper T cells were negatively correlated with IL-6 expression. The infiltration levels of resting mast cells, and M2 macrophages were positively correlated with TNF-α expression; the infiltration levels of follicular helper T cells, and activated mast cells were negatively correlated with TNF-α expression. Spearman correlation between hub IR-DEGs and M0 macrophages and neutrophils that identified key immune cells (Fig. 7F). Therefore, IL-6, TNF-α, IL-1β, CXCL8, and CCL2 may participate in the occurrence and development of pulpitis by regulating the corresponding immune cells, which needs further experimental verification.

### Network construction of hub genes regulated by TFs and miRNAs

**Fig. 8:**
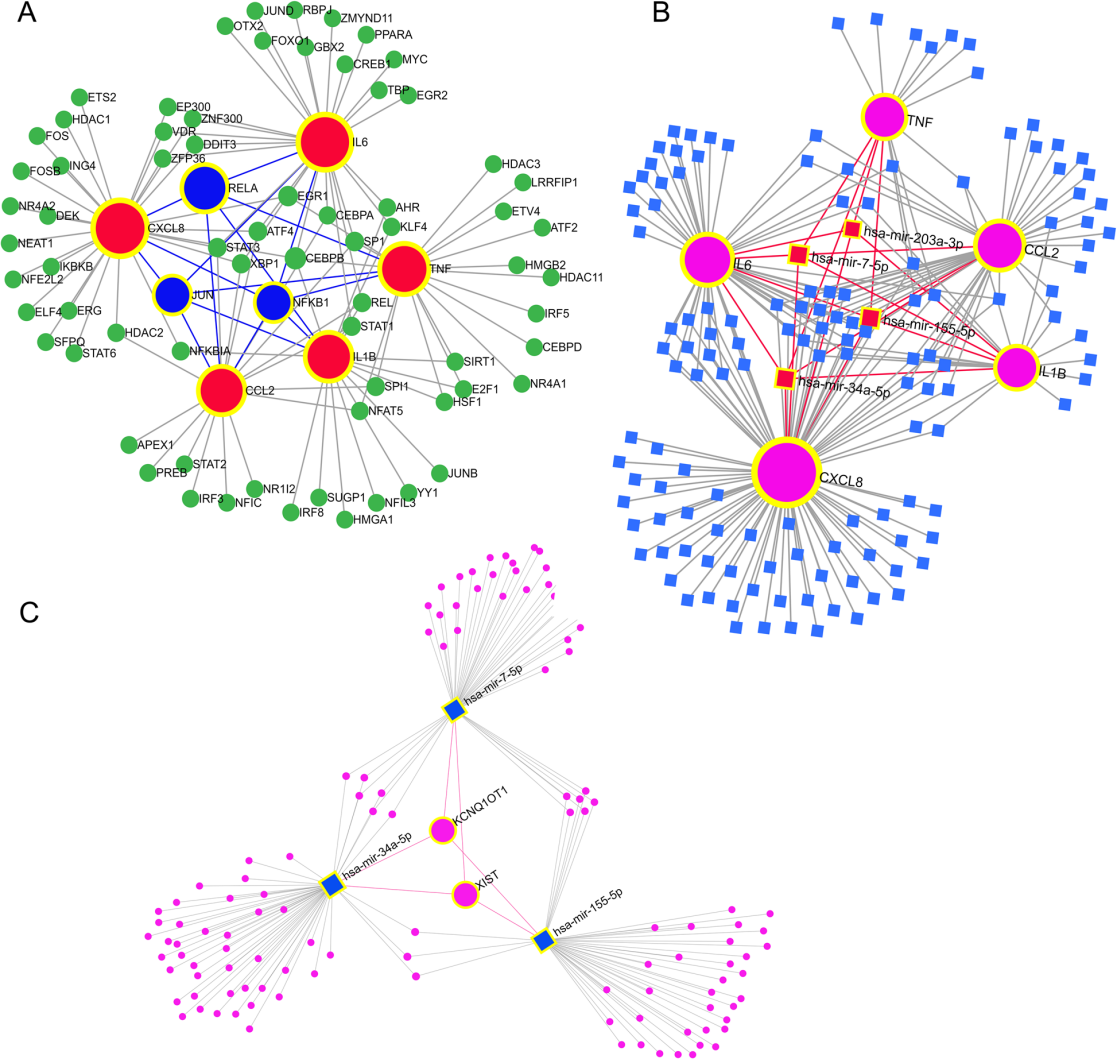
The regulatory network of hub genes. **A** The regulatory network of TFs and hub genes. **B** The interactions between miRNAs and hub genes. **C** The regulatory network of lncRNAs and targeted miRNAs

Based on the miRNet database, a database of interactions with genes and miRNAs, we further constructed a regulatory network of miRNAs and transcription factors for hub genes and found that IL-6, TNF-α, IL-1β, CXCL8, and CCL2 were regulated by multiple miRNAs and transcription factors. Notably, RELA, JUN, and NF-κB1 can regulate five core genes simultaneously (Fig. 8A). Three miRNAs, miR-7-5p, miR-155-5p, and miR-34a-5p can regulate five hub genes simultaneously and may be regulated by two lncRNAs (XIST and kcnq1OT1) (Fig. 8B and C). Finding out how regulators of gene expression might influence disease progression may provide new insights into disease diagnosis and treatment.

## Discussion

In this paper, we first clarified the characteristics of immune cell infiltration in pulpitis and found that neutrophils and M0 macrophage were significantly different immune cells. Next, IR-DEGs were identified and analyzed for functional enrichment and five hub genes (IL-6, TNF-α, IL-1β, CXCL 8, CCL2) were screened. Finally, the correlation between the differentially expressed immune cells and hub genes showed a high correlation, and the regulatory network of hub genes was as follows: four miRNAs (i.e., miR-203a-3p, miR-7-5p, miR-155-5p, miR-34a-5p), and two lncRNAs (i.e., XIST and kcnq1OT1), three transcription factors (i.e., RELA, NFκB1, and JUN), and signal pathways (i.e., TNF-α, NF-κB, JAK-STAT, PI3K-Akt, chemokine, IL-17, Toll-like receptor 2 (TLR2), T cell receptor, and NOD-like receptor). Previous studies also support these results, which we will discuss in the following paragraphs.

### The biological process of IR-DEGs available to participate in pulpitis

An enrichment of IR-DEGs with GO revealed that they chemotactically interact with neutrophils, cytokine-mediated immune response, signaling receptor activator activity, chemokines and receptor activation, and cell membrane receptor activation. This indicates that the microorganism invades the dental pulp tissue to activate receptors on the cell membrane, and chemokines in the cell matrix recruit immune cells to respond to the invasion of the microorganism. KEGG enrichment analysis showed that IR-DEGs were mainly enriched in cytokine-cytokine receptor interaction, chemokine signaling pathway, TNF signaling pathway, NF-κB signaling pathway, and IL-17 signaling pathway. The results showed that the signaling pathways mainly involved in the differential cytokine-cytokine receptor interaction genes appeared in the TNF, NF-κB, and IL-17 signaling pathways. The decrease of NF-κB nuclear translocation may be involved in the immune regulation of primary pulpitis [15, 16]. The combination of microbial ligands and PRRs activates the intracellular signaling pathways of NF-κB and mitogen-activated protein kinases (MAPK), thus releasing antibacterial peptides, cytokines, and other antibacterial components [17].

### Neutrophils and M0 macrophages may be the most important immune cells in the progression of pulpitis

Our results of bioinformatics analysis showed that seven immune cells, resting dendritic cells, resting mast cells, monocytes, M0 macrophages, CD8 T cells, neutrophils, and follicular helper T cells were significantly different in inflammatory dental pulp than in normal tissues, especially neutrophils and M0 macrophages. The release of neutrophil external trap (net) in the dental pulp tissue is a double-edged sword. It can limit bacterial infection, but it may also aggravate cell death and chronic inflammation [18]. It is reported that macrophages play their roles in these processes in various ways [19]. M0 Macrophages in an inflammatory state can differentiate into two phenotypes: pro-inflammatory M1 and anti-inflammatory M2 [20]. Dental pulp fibroblasts can induce M0 macrophage to differentiate into pro-inflammatory M1 in the inflammatory area and have a high bacterial phagocytosis function to control infection. The periphery of the inflammation area can induce macrophages to differentiate into anti-inflammatory M2 cells with repair function. The dynamic balance between them can regulate the inflammation of the dental pulp [21]. Positive and/or negative feedback of cytokine signals received by immune cells and histiocytes determines the extent of cell clonal expansion and the progression of differentiation into effector and memory cells.

### Hub genes mainly regulate the immune response of dental pulp to microorganisms.

We found that IL-6, TNF-α, IL-1β, CXCL8, and CCL2 were co-present in the Top 10 hub genes identified by the five CytoHubba topological methods. Tumor necrosis factor α (TNF-α, TNF) regulates the immune system, cell survival signal pathway, proliferation and metabolic process [22]. An essential inflammatory mediator in pulpitis, TNF-α stimulates the NF-κB signaling pathway to mediate the inflammatory process of pulp tissue [23]. Farges JC et al*.* demonstrated that following interacting with TLR2, odontoblast-like cells generate pro-inflammatory and anti-inflammatory cytokines such as IL-6 and CXCL8 [24]. TLR-2, TLR-4, TLR-9, TNF-α, IL-6, IL-8, IL-17R, IL-23A, NF-κB, MAPK1, DMP1, DSPP, and SOX2 gene expression levels in dental pulp tissue were considerably upregulated in response to inflammatory stimulation [25]. Immune response that is overactive is detrimental. In order to guarantee that the immune response is appropriately regulated, signaling molecules like cytokines have both positive and negative regulatory effects on immune cells [26]. Our results demonstrated IL-6, TNF-α TNF, IL-1β, CXCL8 and CCL2 are closely related to the immune infiltrating cells in pulpitis. In comparison to normal dental pulp samples, irreversible pulpitis samples had considerably higher amounts of IL-1, IL-2, IL-6, IL-8, and TNF-α, which provided a solid foundation for prospective indicators. In particular, IL-6 and TNF-α appeared to be more promising [27]. By producing and secreting TNF-α, IL-1, and CXCL8, immature dendritic cells (DC) contribute to the immune response of human dental pulp to oral pathogens entering dentin during dental caries [28]. Our results are similar to previous experimental results and all demonstrate significant changes in cytokines in different endodontic conditions [29]. Lipoteichoic acid (LTA)-stimulated dental pulp fibroblasts transformed macrophages into the M1 phenotype, which improved their capacity for phagocytosis and resulted in higher TNF-α production [21]. Different pulp capping materials can affect the migration and secretion of IL-1β by human neutrophils. Only MTA can improve the secretion of IL-1β [30]. Mast cells in oral tissues contain TNF-α in their granules, and its release promotes leukocyte infiltration, and the synthesis and release of IL-6 and TNF from mast cells have immunomodulatory effects on CD8 + T cells [31]. Dental pulp stem cells secrete an exosome that prevents CD4 + T cells from differentiating into helper T cells 17 (Th17), lowers the secretion of IL-17 and TNF-α, encourages the polarization of CD4 + T cells into Treg, and releases IL-10 and TGF-β [32]. Cytokines may promote a beneficial or damaging immune response to pulpitis by controlling the migration or polarization of immune cells. In conclusion, we identified five key genes that influence immune cell regulation; however, the precise molecular process still has to be elucidated.

### Influence of microRNA on immunoregulatory network of pulpitis

MiR-7-5p, miR-155-5p, and miR-34a-5p were identified as common targeting microRNAs of the five essential genes by predicting the targeting microRNAs of these genes. We therefore propose that these three microRNAs function as the primary regulators of the immune response to pulpitis. By overexpressing has-miR-7-5p, plasma exosomes derived from septic patients directly inhibit Bad and improve T-lymphocyte apoptosis [33]. Has-miR-7-5p is presumed to be involved in the T cell regulation during the immune response to pulpitis. Several physiological and pathological processes, including immunity, inflammation, virus infection, cancer, and cardiovascular disorders, are significantly influenced by miR-155-5p. It is important to note that miR-155-5p is highly expressed in activated B cells and T cells and monocytes/macrophages [34]. Since pulpitis involves microbial infection, an inflammatory response, and an immunological response, miR-155-5p may be implicated in pulpitis. MiR-34a-5p, a microRNA associated with cell senescence, can be released by exosomes derived from macrophages and result in myocardial cell senescence [35]. Further experimental testing is required to determine whether the macrophages in the dental pulp tissue may induce the cells to enter the aging process by secreting miR-34a-5p. We identified NF-κB, RELA, and JUN as the targeted transcription factors of essential genes. NF-κB is an essential element of the NF-κB signaling pathway and plays an essential role to the pulpitis process. Stress-induced apoptosis in dental pulp cells is mediated through the c-Jun N-terminal kinase (JNK) pathway. Only a small number of dental pulp cells in healthy dental pulp are activated for c-Jun, but JNK is not. However, the injured tooth pulp tissue contained active JNK and c-Jun [36]. Although it has not been investigated, the molecular mechanism of RELA in the progression of pulpitis is also the focus of our future study. Further study of the regulatory network of key genes we screened will help to develop new therapeutic strategies, reduce inflammation of pulp tissue, and possibly promote the development of dentin.

### Limitations

Although this study found the relationship between immune cells and pulpitis, as well as the genes and pathways that may be involved, this study still has some limitations and uncertainties that should be acknowledged. First, the GEO dataset did not contain enough samples, which may lead to statistical errors. Second, the existing dataset contains insufficient general information, making it difficult to consider the different factors. Finally, we haven't verified the key IR-DEGs at the transcription level and protein level, so we need to further explore the molecular expression and its potential mechanism under experimental conditions.

## Conclusion

Gene expression profiles and bioinformatics analysis confirmed that M0 macrophages and neutrophils play an irreplaceable role in pulpitis immunity, and the critical genes in the immune reaction to pulpitis are suggested to be IL-6, TNF-α, IL-1β, CXCL8, and CCL2. Verify that potential role of the NF-κB signaling pathway in pulpitis. A better understanding of pulpitis' immune regulatory network and more effective treatment will result from the findings of this study.

## Data Availability

The datasets used and/or analyzed during the current study are available from the corresponding author upon reasonable request.

## Abbreviations

DEGs: Differentially expressed genes
GEO: Gene expression omnibus
GO: Gene ontology
KEGG: Kyoto encyclopedia of Genes and Genomes
PPI: Protein–protein interaction
IRGs: Immune-related genes
IR-DEGs: Immune-related differentially expressed genes
BP: Biological process
MF: Molecular function
CC: Cellular component
lncRNA: Long non-coding RNA
PVT1: Plasmacytoma variant translocation 1
TFs: Transcription factors
IL-6: Interleukin-6
TNF-αIL-1β: Tumor necrosis factor—alpha
CXCL8: Interleukin-1 betaInterleukin-8
CCL2: Chemokine (CC-motif) ligand 2
NF-κB: Nuclear factor-κB
TLR2: Toll-like receptor 2
MAPK1: Mitogen-activated protein kinase
DMP1: Dentin matrix protein-1
DSPP: Dentin sialophospho protein
SOX2: Sex determining region Y-box 2
